# Immune-Based and Novel Therapies in Variant Histology Renal Cell Carcinomas

**DOI:** 10.3390/cancers17020326

**Published:** 2025-01-20

**Authors:** Justin W. Miller, Jeffrey S. Johnson, Christopher Guske, Gowtam Mannam, Firas Hatoum, Michelle Nassar, Marine Potez, Adnan Fazili, Philippe E. Spiess, Jad Chahoud

**Affiliations:** 1 USF Health Morsani College of Medicine, Tampa, FL 33602, USA; justinmiller1@usf.edu (J.W.M.);; 2 Department of Genitourinary Oncology, H. Lee Moffitt Cancer Center and Research Institute, Tampa, FL 33612, USA; 3 University of South Florida, Tampa, FL 33602, USA; 4 Department of Immunology, H. Lee Moffitt Cancer Center and Research Institute, Tampa, FL 33612, USA

**Keywords:** renal cell carcinoma, non-clear cell, papillary RCC, chromophobe, collecting duct, renal medullary carcinoma, translocation RCC, sarcomatoid

## Abstract

Renal cell carcinoma (RCC) is a heterogeneous disease that represents the most common type of kidney cancer. The classification of RCC is primarily based on distinct morphological and molecular characteristics, with two broad categories: clear cell RCC (ccRCC) and non-clear cell RCC (nccRCC). Clear cell RCC is the predominant subtype, representing about 70–80% of all RCC cases, while non-clear cell subtypes collectively make up the remaining 20–30%. Non-clear cell RCC encompasses many histopathological variants, each with unique biological and clinical characteristics. Additionally, any RCC subtype can undergo sarcomatoid dedifferentiation, which is associated with poor prognosis and rapid disease progression. Recent advances in molecular profiling have also led to the identification of molecularly defined variants, further highlighting the complexity of this disease. While immunotherapy has shown efficacy in some RCC variants and subpopulations, significant gaps remain in the treatment of rare subtypes. This review explores the outcomes of immunotherapy across RCC subtypes, including rare variants, and highlights opportunities for improving care through novel therapies, biomarker-driven approaches, and inclusive clinical trial designs.

## Simple Summary

Kidney cancer, or renal cell carcinoma (RCC), includes several subtypes that require different treatments. While immunotherapy has improved outcomes for the most common type, clear cell RCC, less is known about its effectiveness in rare subtypes. These rare cancers are challenging to study because they are uncommon and are often excluded from clinical trials. This review highlights the challenges in treating these subtypes, explores new research on immunotherapy, and emphasizes the need for more inclusive studies to improve outcomes for all RCC patients.

## 1. Introduction

Renal cell carcinoma (RCC) represents a significant global health concern, with an estimated worldwide incidence nearing 400,000 newly-diagnosed patients each year [1]. Incremental developments in diagnostics and treatment have improved outcomes; however, further reducing the morbidity and mortality of RCC remains an ongoing effort. A major challenge in improving patient outcomes is the heterogeneity of renal cell carcinomas, which encompass a broad spectrum of histological subtypes, each with distinct molecular and clinical characteristics. Beyond the classifications of clear cell RCC (ccRCC) and non-clear cell RCC (nccRCC), the World Health Organization (WHO) recognizes several nccRCC histologic subtypes, including papillary, chromophobe, translocation, collecting duct carcinoma, renal medullary carcinoma, and newly-defined molecular variants [2]. Additionally, RCC tumors may exhibit sarcomatoid histological features, which represent a high-grade, dedifferentiated transformation associated with aggressive tumor behavior [3]. Non-clear cell histologic variants collectively account for approximately 20–30% of RCC cases and are associated with poor prognosis and limited therapeutic options in comparison to clear cell RCC [4]. Additionally, sarcomatoid dedifferentiation is identified in approximately 5% of RCC diagnoses and confers a similarly poor prognosis [3]. Despite their significant clinical burden, investigations into these histologic variants remain underfunded and underrepresented, with the majority of clinical trials excluding or limiting the enrollment of variant histologies.

In ccRCC, clinical trial data have established comprehensive guidelines for the use of tyrosine kinase inhibitors (TKIs), immune checkpoint inhibitor (ICI)/TKI or dual ICI combination regimens, and ICI monotherapy, supported by large, phase III studies such as the CheckMate 214, KEYNOTE-426, CheckMate 9ER, CLEAR, and KEYNOTE-564 trials [5,6,7,8,9]. Furthermore, trials such as CARMENA and SURTIME have added nuance to the role and timing of cytoreductive nephrectomy (CN) in conjunction with systemic therapy in ccRCC [10]. In contrast, the management of variant histology renal cell carcinomas relies on smaller trials, retrospective studies, subgroup analyses from large-phase III trials, and extrapolation from ccRCC data. For example, the KEYNOTE-564 trial, which established the benefit of adjuvant pembrolizumab in ccRCC, excluded patients with non-clear cell histologies, thus limiting the generalizability of the study findings [11]. Nonetheless, some progress has been made in improving the management of variant histology RCC. The Southwest Oncology Group (SWOG) 1500 PAPMET trial, which compared the efficacy of cabozantinib, crizotinib, and savolitinib to sunitinib as first-line therapy for papillary RCC, established a progression-free survival benefit with cabozantinib over sunitinib in this population [12]. Similarly, data from the phase II KEYNOTE-B61 trial indicated that pembrolizumab–lenvatinib combination therapy was more efficacious compared to sunitinib in prolonging survival for patients with nccRCC [13]. Moreover, the CA209-9KU study found that nivolumab–cabozantinib combination therapy was superior to sunitinib in a cohort of 40 patients with nccRCC [14].

Despite these advances, significant questions remain unanswered for variant histology RCC. Critical gaps include defining the role, if any, of cytoreductive nephrectomy, determining the efficacy of adjuvant therapies, and optimizing targeted and systemic treatment strategies across rare subtypes. Addressing these challenges requires dedicated research and the enrollment of patients with variant histologies in larger, more robust clinical trials. This review aims to provide an analysis of immunotherapy in the histopathologic variants of renal cell carcinoma, emphasizing the distinct biology of histologic subtypes, the evolving therapeutic landscape, and residual gaps in evidence that must be addressed to improve outcomes for this underserved population.

## 2. Non-Clear Cell Renal Cell Carcinoma

Non-clear cell RCC cumulatively represents approximately 20–30% of RCC subtypes, and encompasses a wide variety of genetic, histological, and clinical presentations, which has resulted in a lack of robust data to guide therapy selection for individual subtypes [15]. Treatment recommendations for this population have historically been derived from subgroup analyses of large, randomized clinical trials. Enrollment into ongoing clinical trials is often a preferred first-line option for this population in order to build a broader evidence base for the treatment of nccRCC subtypes; however, TKI monotherapy or TKI/ICI combination therapy are also recommended [16]. To date, multiple clinical trials have investigated immunotherapeutic monotherapy and combination regimens in the nccRCC population (Table 1) [17].

**Figure 1:**
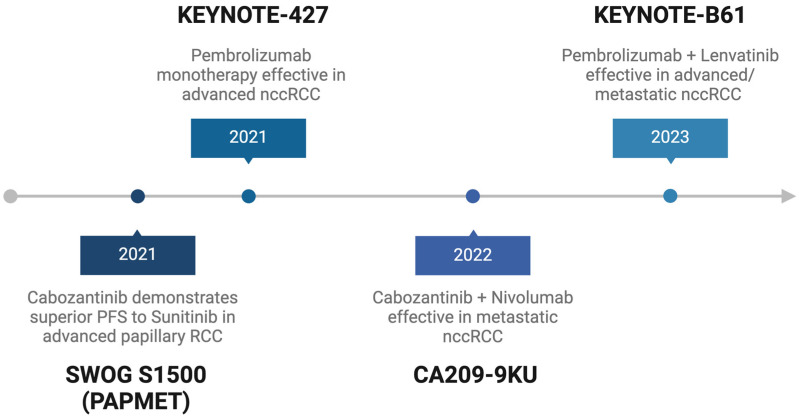
Timeline and summary of results for key clinical trials evaluating first-line immune therapies in non-clear cell renal cell carcinoma (nccRCC). RCC: renal cell carcinoma; SWOG: Southwest Oncology Group.

Additionally, clinical trials have evaluated TKI and ICI monotherapy and combination regimens as first-line interventions in this population (Figure 1, Table 2). For papillary RCC, the PAPMET, KEYNOTE-B61, and CA209-9KU studies each demonstrated improved efficacy compared to the standard-of-care sunitinib. Furthermore, the KEYNOTE-427 study found significant antitumor activity with pembrolizumab monotherapy in advanced pRCC, thus representing a promising option for patients who may not be candidates for targeted therapy or TKI/ICI combinations. In chromophobe RCC, the KEYNOTE-B61 trial reported an objective response rate of 35% (95% confidence interval [CI], 18–54%), highlighting the activity of lenvatinib–pembrolizumab combination in this challenging subtype. Both the CA209-9KU and KEYNOTE-B61 trials have suggested that ICI/TKI combination therapies, such as cabozantinib–nivolumab and lenvatinib–pembrolizumab, may be more effective than sunitinib in unclassified and translocation RCC; however, data remain limited due to the small sample sizes in these cohorts. The KEYNOTE-427 trial demonstrated an objective response rate of 30.8% (95% CI, 20.8–37.9%) in unclassified RCC, highlighting the potential efficacy of pembrolizumab monotherapy in this poorly-characterized subgroup. This notable response rate suggests that immunotherapy may be an effective option to overcome the lack of treatment strategies for this heterogeneous population.

Below, we characterize the major subtypes of non-clear cell RCC and discuss their unique biological and clinical features, as well as emerging data regarding immune targets and therapeutic strategies.

### 2.1. Papillary Renal Cell Carcinoma

#### 2.1.1. Epidemiology and Molecular Features

Papillary RCC (pRCC) is the second most common variant of RCC overall, constituting 10–15% of cases, and is the most common variant histologic subtype [38]. Recent WHO classification guidelines further subdivide pRCC into “classic” papillary RCC and renal papillary adenoma, diverging from the previous type 1 and type 2 classifications of these tumors due to significant clinical and morphological heterogeneity in type 2 pRCC [2]. Several studies have identified a trisomy or partial gain of chromosome 7 as a characteristic feature of pRCC [39,40,41]. *MET* mutation and amplification represent the common molecular features of pRCC, and act to upregulate signaling pathways such as RAS/MAPK, PI3K/AKT/mTOR, and STAT3, promoting cell proliferation and survival [42,43]. In a study of 169 pRCC patients, germline and somatic mutations of the *MET* gene were observed in approximately 33% of patients with type 1 pRCC and 7% with type 2 pRCC [44]. Moreover, Albiges et al. identified *MET* amplification across pRCC tumors irrespective of sub-classification, with increased DNA copies in 86% and 41% of type 1 and 2 pRCC, respectively [45]. Beyond *MET* mutations, alterations in the *FH*, *CDKN2A*, *SETD2*, *TFE3*, and *FLCN* genes have also shown associations with pRCC [46,47]. 

#### 2.1.2. Treatment Paradigms and Emerging Therapies

Papillary RCC has demonstrated a modest response to targeted agents; however, there remains a lack of evidence for treatment, especially in the advanced and metastatic settings [38]. Notably, the completion of clinical trials in this population has been hindered by difficulties in patient recruitment and ensuring consistent definitions of the *MET*-altered status across studies [48]. The PAPMET trial investigated the efficacy of several targeted *MET* inhibitor monotherapies, but only cabozantinib has demonstrated superiority to standard-of-care sunitinib to date [48,49]. The CALYPSO trial studied savolitinib and durvalumab combination therapy and found improvements in OS, progression-free survival (PFS), and objective response rate (ORR) among the subset of patients with *MET* driver mutations, but not in the overall patient cohort. The efficacy of ICIs in pRCC is unclear, with studies of single-agent ICIs in metastatic pRCC reporting ORRs between 8% and 25% [50]. The SAMETA trial is currently underway and seeks to investigate the efficacy of ICI/TKI combination compared with TKI and ICI monotherapy in the *MET*-driven locally advanced/metastatic population [51]. The analysis of the pRCC tumor immune microenvironment (TIME) by de Vries-Brilland et al. lends some insight into its mixed response to immunotherapies. The study identified two distinct immunophenotypes, “immune-enriched” and “immune-low”, with the former group demonstrating increased expressions of the LAG3, TIGIT, CTLA-4, PD-1, and PD-L1 immune checkpoint markers [50]. The stratification of patients based on these immunophenotypes and treatment selection corresponding to checkpoint expression may improve ICI efficacy in patients with immune-enriched pRCC tumors; however, further investigation and validation are needed.

### 2.2. Chromophobe Renal Cell Carcinoma

#### 2.2.1. Epidemiology and Molecular Features

Chromophobe RCC (chRCC) represents the third most common histologic variant of RCC, accounting for approximately 5% of cases [52,53]. Chromophobe RCC tumors may be further categorized into either classic chRCC, eosinophilic chRCC, or mixed-type [52,54]. The immunohistochemical (IHC) staining of chRCC often identifies CK7 and CD117 (also known as C-*kit*) [54,55]. Recent studies have also demonstrated that genetic signatures such as *FOXI1*, *RHCG*, and LINC01187 (a long non-coding RNA) are characteristic of chRCC, and may represent novel biomarkers for these tumors [54]. The unique biological pathways of tumorigenesis in chRCC have yet to be fully described; however, two proposed mechanisms are mutations of the *PTEN* tumor suppressor gene leading to upregulation of the protein mTORC1, which is associated with the cell growth, proliferation, and overproduction/under-neutralization of reactive oxygen species causing cellular oxidative stress [56].

#### 2.2.2. Treatment Paradigms and Emerging Therapies

Following primary treatment with surgical resection, patients with chRCC frequently experience prolonged overall survival (OS), with 5-year OS rates between 78% and 100% [52]. Despite excellent response to surgery, large tumors, extensive necrosis, and sarcomatoid features are associated with worse outcomes in chRCC [52]. Furthermore, unresectable or metastatic chRCC tumors often show very poor response to ICI agents [56]. A systematic review conducted by Msaouel et al. found that the ORR of chRCC across 13 ICI clinical trials was 6.3% [57]. In support of these findings, transcriptomic analyses of chRCC tumors have revealed a lack of immune response present within the tumor, with the few infiltrating immune cells demonstrating reduced checkpoint expression [56]. Additionally, IHC staining for PD-L1 in chromophobe RCC identified positive expression in only 5.6% of tumors [58]. Aside from checkpoint blockade, the use of mTORC1 inhibitors in chRCCs has shown some preliminary success, with a 33% partial response (PR) rate seen with everolimus monotherapy, and an ORR of 44% in early trials of lenvatinib/everolimus combination therapy [56]. Studies investigating novel therapeutic targets have identified hypersensitivity to oxidative stress as an exploitable vulnerability of chromophobe RCC, which suggests a potential role for ferroptotic agents that accumulate iron-dependent peroxides to induce tumor cell apoptosis [56,59,60]. 

### 2.3. Collecting (Bellini) Duct Renal Cell Carcinoma

#### 2.3.1. Epidemiology and Molecular Features

Collecting (Bellini) duct renal cell carcinomas (cdRCCs) are rare tumors arising from the epithelial layer of the distal collecting duct which account for less than 2% of RCC diagnoses [61]. These tumors are highly aggressive, with nearly half of all patients presenting with stage 4 disease, and approximately one third of patients presenting with metastatic disease [61]. The analysis of Surveillance, Epidemiology, and End Results (SEER) database demographics by Wright et al. revealed that Black patients were significantly more likely to be diagnosed with cdRCC than Caucasian patients [62]. The most common genetic mutations associated with cdRCC are *NF2*, *SETD2*, *TP53*, *CDKN2A*, *SMARCB1*, and *MLL* [63]. Interestingly, cdRCC and renal medullary carcinoma have been found to share similar clinical and histopathological features, which may represent an opportunity to leverage treatment strategies and biomarker development from one another to improve outcomes for both populations [64].

#### 2.3.2. Treatment Paradigms and Emerging Therapies

Treatment for unresectable or metastatic cdRCC has primarily involved platinum-based chemotherapy; however, response rates and survival outcomes have generally been very poor [65]. In one study, a combination of platinum chemotherapy with gemcitabine and bevacizumab demonstrated encouraging results, with four of five patients showing reduced tumor burden and one patient achieving a complete response in a solitary metastatic site [66]. Evidence for immunotherapy in cdRCC, however, remains limited. The recently completed BONSAI trial was the first prospective study to evaluate TKI monotherapy in this population, utilizing cabozantinib as a first-line treatment in 23 patients with metastatic cdRCC. The trial achieved its primary endpoint, reporting an ORR of 35%, which is comparable to previous studies of platinum chemotherapy combined with gemcitabine, with or without sorafenib [28]. In addition to these approaches, there is growing interest in identifying alternative molecular targets associated with cdRCC to better treat these patients. Mutations in *NF2*, which have been identified in approximately 30% of cdRCC tumors, have shown sensitivity to mTOR inhibition in studies of other *NF2*-altered tumors [67,68,69,70,71,72]. Based on these findings, Dizman et al. hypothesize that mTOR inhibitors may have therapeutic potential in cdRCC, though further investigation is needed [73].

### 2.4. SMARCB1-Deficient Renal Medullary Carcinoma

#### 2.4.1. Epidemiology and Molecular Features

Renal medullary carcinoma (RMC), recently renamed in WHO guidelines as *SMARCB1*-deficient renal medullary carcinoma, is a rare malignancy that is significantly associated with hemoglobinopathies, particularly sickle cell trait and sickle cell disease [2]. These tumors present most frequently in young males of African descent [74]. Loss of the *SMARCB1* tumor suppressor gene is characteristic of RMC and results in highly aggressive neoplasms; however, detailed mechanistic pathways of tumorigenesis remain unclear [75].

#### 2.4.2. Treatment Paradigms and Emerging Therapies

Therapeutic options for RMC are limited, with platinum-based chemotherapy recommended as a first-line treatment due to the aggressiveness of these tumors [75]. Response to treatment is typically very poor, with a median survival of approximately 14 months [74]. A retrospective study of 10 patients with metastatic RMC found that combination treatment with bevacizumab and erlotinib (VEGF and EGFR inhibitors, respectively) demonstrated potential for use as salvage therapy; however, these findings have yet to be prospectively validated [76]. Efforts to identify novel therapeutic targets are ongoing. For example, through the analysis of RMC cell models, Hong et al. identified a dependent relationship between *SMARCB1*-deficient cells and the ubiquitin–proteasome system, which may represent a targetable pathway for therapeutic intervention in RMC. Furthermore, analysis of the TIME in RMC revealed an abundance of CD4+ and CD8+ lymphocytes, FOXP3+ regulatory T-cells, CD68+ macrophages, and CD20+ B-cells, indicating an immunologically active microenvironment with a combination of pro- and anti-inflammatory elements [77]. Moreover, RMC tissues showed elevated PD-1, CTLA-4, and LAG3 expression, which highlight a potential for ICI efficacy in this population [77].

### 2.5. Translocation Renal Cell Carcinoma

#### 2.5.1. Epidemiology and Molecular Features

Translocation RCC (tRCC) is a rare cancer of the renal proximal tubule epithelium, occurring in less than 5% of adult RCC patients [78]. However, in children and adolescents, tRCC is one of the more common molecular variants, representing approximately 40% of pediatric RCC diagnoses [79]. Translocation RCC tumors are characterized by the fusion of the microphthalmia-associated transcription (*MiT*) and transcription factor E (*TFE*) genes on chromosome Xp11.2 [80]. In particular, the *TFE3* gene is most frequently involved; however, other fusion partner combinations (e.g., *TFE3*, *TFEB*, *TFEC*, *MiTF*) do occur [4]. Alterations in the MiT/TFE family of transcription factors promotes tumorigenesis through the manipulation of cell metabolism, differentiation, and stress adaptation [81]. Notably, tRCC has a wide variety of histological presentations, with a unique capability to mimic almost all other subtypes of RCC, which represents both a challenge and an opportunity to better identify these tumors. In fact, several studies have retrospectively identified tRCC in cohorts of patients originally diagnosed with ccRCC or pRCC [46,82,83]. Translocation RCC tumors were historically diagnosed with IHC staining for *TFE3* or *TFEB* antibodies; however, recent shifts towards the use of fluorescence in situ hybridization (FISH) may help reduce the incidence of false positive or negative results to more accurately identify tRCC [84,85].

#### 2.5.2. Treatment Paradigms and Emerging Therapies

The rarity of tRCC tumors in addition to the heterogeneity of their clinical and histopathological presentations has resulted in difficulties characterizing these tumors and subsequently developing or repurposing immunotherapies. To date, patients with tRCC have demonstrated some benefit from VEGF-targeted therapy as well as cabozantinib, which inhibits MET and AXL in addition to VEGFR [86]. It is important to note, however, that these findings are derived from small, selected patient cohorts as well as retrospective analyses. Data regarding the efficacy of ICI agents in tRCC are sparse, but one preliminary retrospective analysis has indicated that metastatic tRCC tumors respond similarly to clear cell RCC tumors [87]. VEGFR-TKI/ICI combination therapy has also shown efficacy, albeit in a cohort of only two patients [88]. Multiple clinical trials are underway investigating TKI monotherapy and TKI/ICI combination therapies in tRCC [86]. Recent analyses by Bakouny et al. of the molecular and immune landscape of tRCC identified infiltration of unique CD8+ T-cell immunophenotypes that demonstrated increased LAG3 and decreased TIM-3 expression compared with ccRCC, indicating that LAG3-targeted immunotherapy may benefit these patients [80]. Furthermore, the study found that tRCC tumors consistently upregulated the NRF2 transcription factor pathway, which has shown association with resistance to targeted agents such as sunitinib, axitinib, lenvatinib, and temsirolimus [80]. Further investigation is needed; however, these findings suggest that immune checkpoint inhibition may play a key role in the treatment of tRCC.

### 2.6. Unclassified or Other Renal Cell Carcinomas

In addition to the variants described above, there are a number of additional renal tumors identified in the WHO *Classification of Tumors of the Urinary System and Male Genital Organs* as well as unclassified malignancies [89]. For unclassified renal cell carcinoma cases, it is advisable to perform next-generation sequencing (NGS) and *ALK* testing, as these analyses may identify actionable mutations or alterations that can guide targeted therapeutic strategies and improve clinical outcomes. Incorporating these tests into the diagnostic workflow may provide critical insights for personalized treatment approaches. Notably, the 2022 WHO “Blue Book” update is the first to designate molecularly defined renal tumors, such as *TFEB*-altered RCC, *ALK*-rearranged RCC, and *ELOC*-mutated RCC, which cannot be diagnosed solely by assessment of tumor morphology. As sequencing technologies continue to advance, more refined tumor subclassification and biomarker discovery may guide the development of novel targeted strategies for these molecularly defined entities. Furthermore, by identifying distinct immune profiles and molecular alterations, future efforts may expand the existing role of checkpoint inhibitors and other immunotherapies in these rare and previously unclassified variants, ultimately improving patient outcomes across the broad spectrum of RCC subtypes.

## 3. Renal Cell Carcinoma with Sarcomatoid Features

The presence of sarcomatoid features in renal tumors, characterized by pleomorphic and spindle-shaped cells with high cellularity and pronounced atypia, confers a poor prognosis, with survival durations often lasting less than one year [3]. While the process of sarcomatoid dedifferentiation is often considered to be a rare occurrence in renal cell carcinoma, reported rates of sarcomatoid histological features in the literature have varied from 5% to over 20% [3,90]. Surgical resection represents a first-line treatment option for patients with localized disease; however, approximately 80% of patients with sarcomatoid RCC (sRCC) that undergo nephrectomy have disease recurrence within two years [91,92]. Patients with sRCC often have metastatic disease upon initial presentation, for which cytoreductive nephrectomy and subsequent interferon alfa immunotherapy have demonstrated efficacy in prolonging survival over immunotherapy alone, though there is controversy regarding the delayed initiation of systemic treatment in the current era of immune checkpoint inhibitor therapies [3,93,94].

Treatment guidelines for systemic therapy in sRCC are limited and based on adapted ccRCC guidelines in combination with small single-arm trials, the subgroup analyses of phase III studies, and retrospective chart review studies [95]. TKI monotherapy and chemotherapy/TKI combinations have shown little efficacy in this population, likely due to the aggressive nature, unique molecular characteristics, and immunosuppressive microenvironment of these tumors [95,96,97]. Conversely, dual ICI therapy and ICI/TKI combination regimens have demonstrated promising results in sRCC subgroup analyses. The CheckMate 214, IMmotion151, JAVELIN RCC 101, KEYNOTE-426, CheckMate 9ER, and CLEAR trials each investigated ICI-based combinations compared with sunitinib and found improved outcomes with the use of the checkpoint blockade (Table 3).

Tumors with both non-clear cell histology and sarcomatoid dedifferentiation are highly aggressive and demonstrate significantly worse survival outcomes than clear cell RCC with sarcomatoid features (sarcomatoid ccRCC vs. nccRCC: OS hazard ratio [HR] = 0.13; 95% CI: 0.04–0.44, *p* = 0.0009) [104]. Data regarding the effectiveness of ICI therapies in RCC tumors with non-clear cell histology and sarcomatoid features are sparse. Subgroup analyses from the KEYNOTE-427, NCT02724878, and CheckMate 920 studies have explored the efficacy of first-line immunotherapy in this population (Table 4). Additionally, in a 2023 American Society of Clinical Oncology (ASCO) abstract, Labaki et al. conducted a retrospective review of the International Metastatic Renal Cell Carcinoma Database Consortium (IMDC) database to assess the efficacy of first-line ICI in sarcomatoid/rhabdoid nccRCC and found significantly improved survival compared with VEGF-TKIs (median OS not reached vs. 7.1 months; median time to treatment failure 9.4 vs. 2.9 months) [105].

Investigations into sRCC tumor biology have substantiated evidence for the efficacy of ICI agents in sarcomatoid RCC. Gupta et al. found that RCC tumors with sarcomatoid features exhibited amplified gene expression at the 9p24.1 locus, which harbors the *JAK2*, *PD-L1*, and *PD-L2* genes [106]. Moreover, the amplification of *JAK2* has shown an association with greater PD-L1 expression across several cancers [107,108,109]. These findings concur with IHC analyses of sRCC tumors which identified the increased expression of PD-L1 [3]. Additionally, microenvironment analyses of these tumors revealed significantly greater infiltration of CD8+ T-cells in sRCC compared to RCC without sarcomatoid features [97]. These results highlight the active immune microenvironment of sarcomatoid RCC, supporting the rationale for immune checkpoint inhibitors as a promising therapeutic strategy in this aggressive variant.

## 4. Additional Considerations and Future Directions

**Figure 2:**
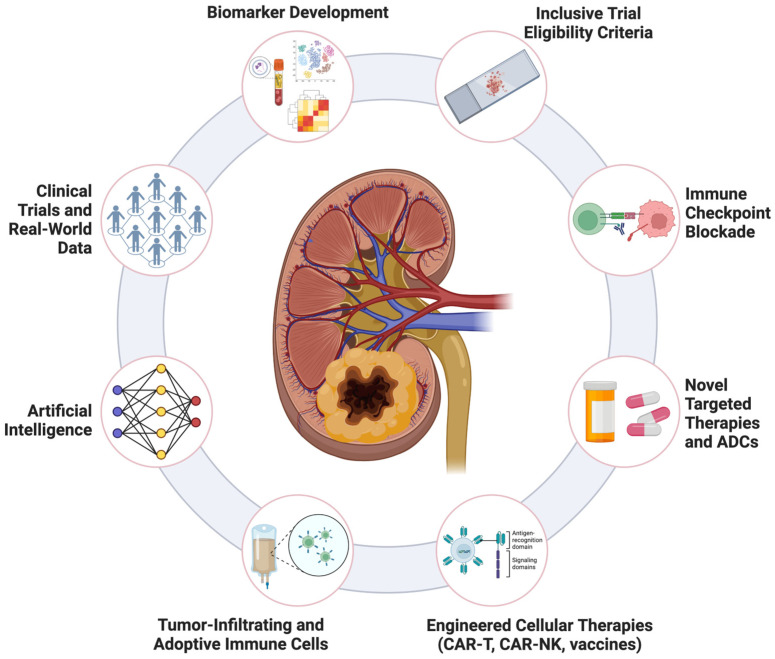
An overview of key strategies shaping the landscape of variant histology renal cell carcinoma treatment. These include advancements in biomarker development, inclusive clinical trial eligibility criteria, immune checkpoint blockade, novel targeted therapies and antibody–drug conjugates (ADCs), engineered cellular therapies (including CAR-T, CAR-NK, and cellular vaccines), tumor-infiltrating and adoptive immune cells, artificial intelligence integration, and the incorporation of real-world data into clinical trials. These interconnected approaches aim to optimize outcomes across diverse histologic subtypes and patient populations.

The evolving therapeutic landscape for variant histology renal cell carcinoma encompasses multiple interconnected approaches including diagnostics, therapeutics, and clinical implementation strategies (Figure 2). Addressing racial and ethnic disparities in treatment and outcomes, advancing precision medicine approaches, and developing novel therapeutic strategies have emerged as crucial considerations for the management of histologic variants.

### 4.1. Renal Cell Carcinoma Histology Distribution and Immunotherapeutic Outcomes Among Racial and Ethnic Minorities

Patient outcomes across cancers, as well as in RCC specifically, appear to depend, at least in part, on race and ethnicity. In a pan-cancer analysis, Shaw and Zhang et al. found that after adjusting for age, economic, and clinical factors, Black patients had worse cancer-specific survival (CSS) in most cancer sites compared to other racial groups [110]. While access to healthcare, socioeconomics, and other systemic factors likely all play a role, genetic differences may also contribute to poor prognoses. The analysis of ccRCC tumor somatic mutations in The Cancer Genome Atlas (TCGA) database revealed that African American patients were significantly less likely to have *VHL* mutations and were more likely to express genes associated with upregulated epithelial–mesenchymal transition (EMT), cell differentiation, and TGF-β signaling [111]. Patients with this clear cell type B (ccB) phenotype were found to have shorter median survival times by a factor of nearly four and a half [112]. These phenotypic differences in patient outcomes demonstrate the importance of considering race and ethnicity when determining treatment strategy. As emerging data continue to clarify how genetic alterations influence tumor immunogenicity and checkpoint expression, a more personalized approach to immunotherapeutic interventions may help mitigate disparities in outcomes.

**Figure 3:**
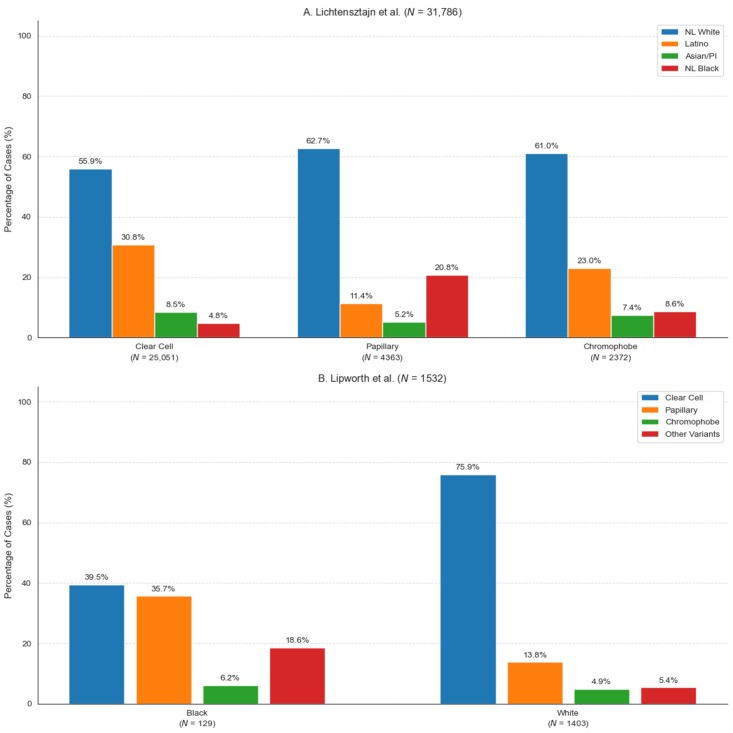
Distribution of RCC histologic variants by race/ethnicity from two independent cohorts. (**A**) Lichtensztajn et al. analyzed 40,016 RCC cases from the California Cancer Registry, showing racial/ethnic distribution within histologically confirmed clear cell renal cell carcinoma (ccRCC, *N* = 25,051), papillary RCC (*N* = 4363), and chromophobe RCC (*N* = 2372) cases. The remaining cases were classified as “other” variants or were not otherwise specified [113]. (**B**) Lipworth et al. analyzed 1532 consecutive cases from a single academic medical center demonstrating the distribution of RCC variants between Black and White patients. The ‘Other Variants’ category includes collecting duct carcinoma (CDC, 3.1% in Black vs. 0.3% in White patients), mixed RCC, unclassified RCC, translocation RCC (tRCC), and other rare variants [114]. NL = Non-Latino; PI = Pacific Islander. Sample sizes (*N*) for each racial/ethnic group are indicated in parentheses.

Studies have also demonstrated that RCC histologic variant distributions are unique across different races and ethnicities (Figure 3). For example, a study of 40,016 RCC cases in the California Cancer Registry found that Latino and Asian American or Pacific Islander patients were significantly less likely than non-Latino White patients to have either papillary or chromophobe RCC in comparison to clear cell RCC [113]. In contrast, the same study found that Black patients were significantly more likely to have pRCC or chRCC than ccRCC in comparison to non-Latino White patients upon multivariate analysis [113]. These findings are corroborated by Lipworth et al. who similarly found that Black patients had a greater proportion of papillary and chromophobe RCC cases compared to Caucasian patients [114]. Interestingly, older Caucasian males are the most common demographic diagnosed with sarcomatoid RCC [115]. Renal medullary carcinoma, a rare and highly morbid histologic variant, occurs almost exclusively in individuals with sickle cell trait (but has been observed in patients with other hemoglobinopathies), which disproportionately affects approximately 8% of Black individuals in the United States [113,116]. These findings present an opportunity to recontextualize the perceived rarity of RCC subtypes by acknowledging the significant fluctuations in prevalence between racial and ethnic groups. This understanding reflects a need to design clinical trials that evaluate immunotherapeutic efficacy in more diverse and representative patient populations.

Encouragingly, there have been improvements in survival across all patient demographics following the adoption of targeted immunotherapy and ICIs [117]. However, recent studies indicate that disparities in outcomes between some populations remain. Guram et al. found no significant differences in OS between Hispanic and non-Hispanic patients with advanced RCC receiving systemic therapy [118]. Similarly, Quinn et al. saw no difference in DFS in a subgroup analysis of Asian and non-Asian patients with RCC treated with adjuvant axitinib [119]. Analysis of the American Social Security Administration (SSA) life expectancy (LE) tables matched with patient cohorts from the SEER database demonstrated that LE predictions for Caucasians, Hispanics or Latinos, and Asians with localized T1 RCC were generally accurate [120]. However, for African Americans, LE estimates were markedly higher than the SEER cohort’s OS, and this OS was the lowest of all racial groups [120]. Furthermore, Rahman et al. studied a cohort of 15,407 patients with metastatic RCC (mRCC) that were insured through Medicare and found that, after adjusting for other characteristics, Black patients were approximately 20–25% less likely to receive oral anticancer treatments, immunotherapy, or any anticancer treatment at all in comparison to White patients [121]. Discrepancies in immunotherapy utilization may stem from the social determinants of health, implicit biases in treatment, or financial strain beyond Medicare coverage; however, further investigation is needed. 

Although these findings reflect significant progress in RCC treatment, disparities in healthcare accessibility, survival outcomes among racial and ethnic minorities, and the unique distribution of histological subtypes underscore the need for more inclusive and equitable treatment guidelines. Bridging these gaps will require continued efforts to improve diversity in clinical trials, further investigation of novel biomarkers, and enhanced accessibility to immunotherapeutic treatment. 

### 4.2. Clinical Trial Design

A persistent challenge in the design and operation of clinical trials is maintaining a balance between maximizing study recruitment, ensuring diverse and representative patient populations, and demonstrating meaningful clinical benefit, while simultaneously maintaining rigorous scientific standards and ensuring feasibility in real-world settings. While there is a growing effort to design more inclusive and adaptive studies, findings from clinical trials may lack generalizability to real-world patients, particularly those with rare subtypes, complex disease presentations, or from underrepresented demographics [122,123,124].

In RCC, and across oncology studies broadly, trial eligibility criteria often exclude patients with variant histopathology and comorbid conditions. A fundamental challenge in studying RCC histologic variants is their inherently low prevalence, which limits patient accrual and often results in underpowered analyses that make it difficult to draw definitive conclusions about optimal treatment approaches. This challenge is further compounded by restrictive clinical trial eligibility criteria. Gan et al. retrospectively assessed the IMDC and Alberta Immunotherapy databases and found that approximately 27% of RCC patients in these databases were ineligible for first-line immunotherapy trials, with the most common disqualifying criteria being non-clear cell histology, anemia, brain metastases, reduced kidney function, poor performance status, and thrombocytopenia [125]. Notably, about one third of trial-ineligible patients were excluded due to failure to meet histopathological criteria alone, indicating a need for broader eligibility criteria that better represent the heterogeneity of RCC subtypes [125]. In recent years, joint stakeholders such as the U.S. Food and Drug Administration (FDA), ASCO, and Friends of Cancer Research have recommended re-assessment of clinical trial eligibility criteria in an effort to improve the generalizability of study results and prevent the unnecessary exclusion of patients that may benefit from novel therapies [126,127,128]. Potential solutions to address the significant clinical trial design gaps for variant RCC histologies may include the systematic inclusion of these variants in dedicated cohorts within larger trials, as well as basket/umbrella trial designs based on shared biomarker profiles (e.g., *NF-2*-altered, *ALK*-rearranged, or *SMARCB1*-deficient tumors) [127,129]. These efforts to expand trial eligibility have been complemented by increased attention to real-world evidence generation.

Real-world data (RWD) derived from patient registries and medical records has increasingly been utilized to assess the efficacy of immunotherapy in patient populations seen in everyday clinical practice [130,131,132,133,134]. As such, it reflects a broader spectrum of clinical scenarios, histologic subtypes, comorbidities, treatment patterns, and patient outcomes than those typically captured in clinical trials. Efforts to better characterize real-world patients and clinical practices are currently underway in the setting of mRCC with the ODYSSEY RCC registry, a phase IV study designed to longitudinally assess evolving treatment paradigms and patient outcomes in the United States [135]. Beyond registry studies, initiatives to incorporate real-world data into clinical trials as external control arms have been explored in hematologic cancers and may represent an approach to enhance the generalizability of results in solid tumor studies as well [136].

### 4.3. Advancing Precision Medicine for Rare Variants

Advancing diagnostic and therapeutic options for histologic variants of RCC has long posed a challenge; however, innovative technologies enable the more detailed and precise characterization of tumors, which may accelerate progress in this field. For example, Li et al. performed multi-omics analyses of both nccRCC and ccRCC tumors to better characterize and compare the unique biology that differentiates subtypes [137]. Additionally, genomic analysis of nccRCC tumors by Carlo et al. found that 15% of tumors harbored potentially targetable somatic mutations, and 9% had somatic mutations for which FDA-approved drugs (in RCC or other indications) are currently available [138]. These findings lay the critical groundwork for deepening our understanding of genetic and epigenetic pathways that contribute to the aggressiveness and therapeutic resistance of rare variants and identifying personalized candidate therapies to better treat patients with these tumors.

Furthermore, the integration of emerging assays such as liquid biopsies may lead to earlier tumor detection, improved treatment selection, and more accurate survival prognostication. To date, liquid biopsy is not an approved diagnostic tool for RCC; however, it represents a promising method to assess tumor characteristics in real-time with minimally invasive techniques, and has received approval for use in lung, breast, prostate, and colon cancers [139]. Preliminary findings regarding the role of liquid biopsies in RCC indicate that extracellular vesicles, exosomes, circRNAs, lncRNAs, and piRNAs hold promise as potential molecular markers, but additional research is needed to identify and validate candidate biomarkers and develop standardized assays [139,140].

Advancements in artificial intelligence (AI) may also contribute to developments in diagnosis, treatment, and survival prognostication. The integration of AI and digital pathology allows for the automated analysis of histological images, which may help reduce false negatives through pre-screening, and can identify subtle features that may play a larger role in tumor behavior [141]. Neural network models have demonstrated impressive accuracy in classifying tumor histology, with reported area under the curve (AUC) values up to 0.98 [142,143,144,145]. Incorporating these models into routine clinical workflows may assist pathologists by identifying regions of importance on digitized slides and providing a reliable second opinion to support clinical decision making. 

AI has also been applied in the field of radiomics, which utilizes algorithms to extract data from medical imaging [146]. For example, Uhlig et al. developed a machine learning algorithm to perform radiomics analyses of RCC tumor computed tomography (CT) images, achieving a moderate ability (AUC = 0.75) to identify clear-cell, papillary, and chromophobe subtypes from imaging alone [147]. Kocak et al. developed a similar radiomics model and found that it performed best when distinguishing clear cell RCC from non-clear cell RCC (external validation accuracy, sensitivity, and specificity of 84.6%, 69.2%, and 100%, respectively), but found difficulty in differentiating individual histological subtypes [148].

Beyond diagnostics, AI’s ability to analyze large datasets can contribute to personalized treatment plans by identifying patterns among patient data and predicting therapeutic responses. Several AI models have been developed to forecast survival outcomes and recurrence risks in RCC patients, demonstrating the promise of these tools in clinical practice [149,150]. Despite rapid progress in the field of artificial intelligence, several barriers remain including the integration of diverse data types, the availability of high-quality and sufficiently large datasets, the interpretability of complex models, and the need for novel approaches to enhance precision and clinical applicability [151].

The utilization of advanced surgical methods including novel minimally invasive techniques, intraoperative ultrasound-guidance, and three-dimensional tumor modeling may also contribute to more personalized care that improves patient outcomes. Robot-assisted partial nephrectomy has emerged as a valuable tool for treating complex cases, allowing for better visualization of tumors and improved instrument articulation, which enhances surgeons’ ability to resect tumors with unusual growth patterns or in challenging anatomical locations [152,153,154]. Furthermore, intraoperative ultrasound-guidance has become increasingly sophisticated, and its use has demonstrated improvements in laparoscopic surgical outcomes including operative duration, blood loss, and ischemia time [155]. Finally, for rare tumors for which prior surgical experience is limited, surgical planning tools utilizing three-dimensional modeling may lead to improved pre-operative assessment and surgical approach selection [156,157,158].

### 4.4. Novel Therapies and Clinical Trials 

The therapeutic landscape for variant histology renal cell carcinomas is rapidly evolving, with a number of clinical trials underway in this space (Table 5). These advancements are driven by an improved understanding of the molecular pathways and immune microenvironments unique to histologic variants. Emerging therapies in RCC, including novel immune checkpoint inhibitors, antibody–drug conjugates, adoptive immune cells, engineered cellular therapies, cellular vaccines, and cytokine/immune-activated cells, represent promising treatment modalities for potential adoption in rare histologic subtypes. While these emerging strategies show promise, further clinical studies are needed to validate their efficacy and safety across RCC histologic variants, which remain an area of unmet clinical need. Below, we discuss several of these innovative approaches and their preliminary results.

#### 4.4.1. Immune Checkpoint Blockade

Several novel immune checkpoints have emerged as candidate targets for therapeutic intervention. The T-cell immunoglobulin and ITIM domain (TIGIT) is a co-inhibitory receptor expressed on various immune cells, including T-cells and natural killer (NK) cells [159]. The analysis of human renal cancer cell expression data indicated that chromophobe and papillary tumors express slightly more TIGIT than controls; however, this result was not statistically significant [159]. Interestingly, Perales et al. found that TIGIT expression in RCC is associated with higher tumor grade and stage and is inversely correlated with PD-1 and LAG3 expression [160]. Notably, this study included both clear cell and non-clear cell RCC tissue samples. The relationship between TIGIT and PD-1/LAG3 expression suggests that TIGIT may serve as a potential biomarker to identify patients who are less likely to benefit from PD-1 or LAG3 inhibition and who may respond to anti-TIGIT therapies. Further validation of this relationship could support a biomarker-driven strategy for more personalized immunotherapy. The ongoing phase Ib/II MK-3475-03A study is investigating the anti-TIGIT agent vibostolimab in combination with pembrolizumab and belzutifan (HIF-2α inhibitor) in advanced clear cell RCC, and results from this trial may provide valuable insights into combination therapy with this novel agent [161].

Lymphocyte activation gene 3 (LAG3) is an additional inhibitory receptor associated with cellular immune regulation and tumor immune evasion [162]. RCC tumors have demonstrated an elevated LAG3 expression, with notable upregulation observed in histologic variants such as papillary RCC, *SMARCB1*-deficient renal medullary carcinoma, and translocation RCC [50,77,80,163]. Clinical trial NCT05347212 is ongoing and aims to study the combination of nivolumab, an anti-PD-1 monoclonal antibody (mAb), and relatimab, an anti-LAG3 mAb. Supporting this clinical focus, preclinical findings by Zelba et al. demonstrated that PD-1 blockade increases LAG3 expression in RCC tumors in vitro [164]. Furthermore, the dual blockade of PD-1 and LAG3 restored T-cell function across ccRCC, sarcomatoid-differentiated ccRCC, and chRCC tumor samples [164]. These findings suggest that LAG3 may serve as a key mechanism of immune resistance in RCC and highlight the potential therapeutic benefit of dual PD-1/LAG3 blockade, particularly in tumors with elevated LAG3 expression or tumors unresponsive to existing immunotherapies.

The T-cell immunoglobulin and mucin-domain containing-3 (TIM-3) checkpoint receptor has been implicated in T-cell exhaustion and tumor immune evasion [165]. A study of RCC tumor samples, which included both nccRCC (28.9%) and sarcomatoid (19.4%) histologies, detected TIM-3 expression in 56.4% of all samples, indicating that it is a feasible target across histologic subtypes [166]. Granier et al. investigated the expression of PD-1 and TIM-3 on CD8+ T-cells and found that co-expression correlated with aggressive tumor phenotypes and poor survival outcomes across RCC tumor histologies [167]. Furthermore, they found that the inhibition of both checkpoints restored cytotoxic T-cell function, and that VEGF upregulation enhanced the expression of both PD-1 and TIM-3 [167]. These findings suggest that anti-TIM-3 agents may show utility in combination with PD-1 inhibition to enhance anti-tumor immune responses and improve outcomes in aggressive RCC variants. A phase I study of a TIM-3 mAb (LY3321367) as a monotherapy or in combination with an anti-PD-L1 agent demonstrated no dose-limiting toxicities; however, minimal anti-tumor activity was observed, especially in tumors refractory to anti-PD-1/PD-L1 agents [168]. The continued exploration of therapeutic regimens targeting TIM-3 may identify new strategies to overcome treatment resistance.

Immunoglobulin-like transcript 4 (ILT4) is an immunosuppressive molecule predominantly expressed in myeloid cells, including monocytes, macrophages, dendritic cells, and granulocytes [169]. While the precise tumorigenic mechanisms of ILT4 are unclear, preliminary data indicate that the inhibition of ILT4 induces a shift from anti-inflammatory M2 macrophages towards pro-inflammatory M1 macrophages in the tumor microenvironment [170,171]. Chen et al. found that the co-inhibition of ILT4 and PD-L1 demonstrated greater efficacy than PD-L1 inhibition alone in NSCLC, but, interestingly, this effect was negated in EGFR-mutant tumors [172]. The first-in-human NCT03564691 study investigated an anti-ILT4 mAb (MK-4830) as a monotherapy and in combination with pembrolizumab in advanced solid tumors [173]. No DLTs were observed, and the combination arm demonstrated modest activity, even in patients who were previously unresponsive or refractory to anti-PD-1/PD-L1 therapy [173]. These findings reflect the potential of ILT4 as a therapeutic target; however, further investigation is needed.

#### 4.4.2. Novel Targeted Therapies and Antibody–Drug Conjugates

CDK4/6 inhibitors such as palbociclib, ribociclib, and abemaciclib target the cell cycle by blocking the transition from the G1 to S phase, thereby inhibiting tumor cell proliferation. Alterations in *CDKN2A* and *CDKN2B*, such as mutations or deletions, are common in RCC and are linked to poor survival across multiple histologies, including clear cell, papillary, collecting duct, and chromophobe RCC [63,174]. The NCT05665361 trial is currently recruiting patients with advanced papillary RCC to investigate the combination of the anti-PD-1 agent sasanlimab with the CDK4/6 inhibitor palbociclib [175].

EZH2 inhibitors have demonstrated promise for RCCs with *INI1* (*SMARCB1*) loss, such as renal medullary carcinoma. *INI1* is a tumor suppressor gene that normally acts to suppress EZH2 activity [176]. In RCC, EZH2 promotes tumor progression through various mechanisms, including the repression of E-cadherin, which enhances cell migration and invasion [177]. EZH2 also plays a role in the activation of STAT3 signaling, which is involved in tumor cell migration, invasion, and angiogenesis [178]. A phase II trial studying the EZH2 inhibitor tazemetostat in patients with *SMARCB1*-deficient renal medullary was recently completed, and results from this study will help inform future treatment approaches for this population [179].

Proteasome inhibitors, such as bortezomib and carfilzomib, are of growing interest in the treatment of RCC. While data regarding the efficacy of proteasome inhibition in variant histology are sparse, bortezomib has shown promise in preclinical and translational studies of ccRCC [180,181,182,183]. Interestingly, Abt et al. found that the addition of human immunodeficiency virus (HIV)-protease inhibitors to carfilzomib in vitro improved cytotoxicity against ccRCC, suggesting a need for further investigation of combination regimens [184].

Antibody–drug conjugates (ADCs) are composed of a monoclonal antibody, chemical linker, and cytotoxic payload, allowing for the targeted delivery of potent anticancer agents [185]. Early trials have begun to investigate the safety and preliminary efficacy of these agents in RCC. A sequential phase I study of two anti-ENPP3 ADCs (AGS-16M8F and AGS-16C3F) demonstrated activity in treatment-refractory metastatic clear cell and papillary RCC tumors [186]. Three of thirteen patients (23%) in this trial exhibited a durable partial response of over two years with AGS-16C3F. Another investigation studied an ADC targeted at TIM-1, which is highly expressed in ccRCC and pRCC tumors [187]. A phase I trial with 16 patients demonstrated promising antitumor activity; however, the trial closed early due to termination of development of the drug [187]. Furthermore, studies of CD70 as an ADC target indicate that it may hold benefit for patients with variant histology. IHC staining for CD70 across RCC histologies identified the expression in ccRCC, pRCC, cdRCC, and sRCC, with sarcomatoid and clear cell RCC demonstrating the highest expression levels [188]. A phase I trial of an ADC targeting CD70 demonstrated a modest response in patients with metastatic RCC; however, the findings warrant the further exploration of these agents [189].

#### 4.4.3. Tumor-Infiltrating and Adoptive Immune Cells

Adoptive cell transfer (ACT) therapy with tumor-infiltrating lymphocytes (TILs) has shown promising results in several cancers, but its use in RCC has previously been limited by inconsistent ex vivo TIL expansion and poor cytotoxic activity [190]. Improvements in RCC TIL manufacturing have led to a resurgence of efforts to leverage this treatment modality. Recent work by Potez et al. demonstrated the feasibility of growing TILs across ccRCC, pRCC, and unclassified histologies (uRCC), with expanded T-cells from pRCC and uRCC demonstrating a greater proportion of CD4+ T-cells compared to ccRCC tumors [191]. Interestingly, this abstract also found that the exposure of RCC TILs to hypoxic conditions (5% O_2_) significantly enhances the differentiation of CD69+CD103+ tissue resident memory T-cells in the TIL products [191]. In contrast to chimeric antigen receptor T-cells (CAR-T), which are engineered to target a single tumor antigen, TILs are inherently polyclonal and harbor multiple T-cell receptor (TCR) clones [192]. This diversity enables TILs to recognize and bind a broader array of tumor antigens that are specific to the unique tumor microenvironment. TCR polyclonality in TILs is particularly advantageous in RCC where tumors are highly heterogenous and comprise a diverse spectrum of histopathological subtypes. Moreover, the lack of effective systemic immunotherapies for rare histologic subtypes such as *SMARCB1*-deficient renal medullary carcinoma and collecting duct RCC represents an opportunity to explore the role of TIL therapy and other novel adoptive immune agents in these populations.

γδ T-cells, a subset of T-cells that recognize tumor cells through major histocompatibility complex (MHC)-independent mechanisms, have also shown early efficacy in early clinical trials of RCC, non-small cell lung cancer (NSCLC), melanoma, and breast cancer [193,194,195]. The analysis of tumor-resident Vδ2− γδ T cells indicated that these cells retain effector function despite displaying exhausted phenotypes, which may promote improved treatment efficacy when used alongside ICI agents [196]. Additionally, lymphokine-activated killer (LAK) cells, T- and NK-cells expressing markers such as CD3-CD56 + and NKG2D, have shown mixed efficacy in small trials, but have largely been superseded by more precisely-targeted cellular therapies [197].

#### 4.4.4. Engineered Cellular Therapies

Chimeric antigen receptor (CAR)-T cell therapy utilizes genetically modified T-cells engineered to express CARs targeting tumor-specific antigens. Rare histologic subtypes, such as *SMARCB1*-deficient renal medullary carcinoma, translocation RCC, and collecting duct carcinoma, often express distinct biomarkers that are less prevalent in ccRCC. As a result, CAR-T cells can be engineered to target unique antigens, providing a more personalized approach to therapy for these patients. Multiple clinical trials are underway assessing CAR-T cells with different molecular targets in RCC [198]. Among these, CD70-targeted CAR-T therapies have shown promising results in clinical trials for advanced ccRCC to date [199]. The phase I COBALT-RCC trial, which assessed CD70-targeted CAR-T cells in heavily pretreated ccRCC, reported a disease control rate (DCR) of 81%, indicating that CAR therapy may play a role in overcoming ICI/TKI treatment resistance [200]. High expression levels of CD70 have been demonstrated in RCC with sarcomatoid differentiation, and modest expression levels were seen in pRCC and cdRCC, which highlights the potential of the use of these agents in variant histology RCC [188].

CAR-NK cells are also being studied in RCC as an additional therapeutic approach. Zhang et al. demonstrated the in vitro activity of CAR-NK-92 cells alone and both the in vitro and in vivo efficacies of CAR-NK-92 in combination with cabozantinib [201]. CAR-NK cells offer unique advantages over CAR-T cells, including a lower risk of cytokine release syndrome and graft-versus-host disease, and the ability to function in a MHC-independent manner, making them particularly suitable for heterogeneous and immune-evasive tumors such as RCC [202].

Preclinical investigations into CAR designs combining immune checkpoint blockade with T-cell activation have shown promise for future use in RCC. For example, T-cells with chimeric PD-1:28 receptors restored type 1 T helper (Th1) cell function in the immunosuppressive RCC TIME without activating Th2 cells [203]. Furthermore, chimeric PD-1:28 T-cells in combination with ICI secreted significantly more IFNγ than T-cells without chimeric PD-1:28, indicating a potential benefit of combination therapy regimens [203].

Future directions for CAR therapy in RCC include the discovery and utilization of novel tumor antigens specific to histological variants, the optimization of CAR constructs to improve binding and reduce off-target toxicity, and the incorporation of combination regimens with systemic therapies to enhance treatment efficacy. Additionally, characterizing the unique molecular and immune profiles of histologic variants may reveal new targets suitable for CAR-based approaches, filling a critical gap in treatment modalities for this population. While employing these therapeutic approaches represents an important opportunity, several significant challenges exist. The most notable barriers are the substantial cost of conducting industry-sponsored clinical trials for rare subtypes and difficulties with trial recruitment. However, potential solutions include collaborative funding models that incorporate support from federal resources, advocacy organizations, philanthropic foundations, and academic institutions. Furthermore, the implementation of centralized, referral-based trial networks could optimize patient accrual and enable the conduct of meaningful clinical studies despite the rarity of these variants.

#### 4.4.5. Cellular Vaccines

Personalized vaccines using autologous dendritic cells (DCs) loaded with tumor antigens represent an innovative strategy to prime T-cells against cancer. A Phase I/II trial demonstrated that vaccination with DCs containing either telomerase and HLA-A2 binding peptides or tumor lysate in combination with IL-2 resulted in stable disease for 8 weeks in 48% of patients with mRCC [204]. Furthermore, a recent abstract found that DCs pulsed with Profilin-1 (Pfn1) peptides slowed the growth of subcutaneous RCC tumors in murine orthotopic models and thus may hold promise as a monotherapy or ICI-combination regimen [205].

#### 4.4.6. Cytokine and Immune-Activated Cells

Cytokine-induced killer (CIK) cells are T- and NK-cells activated and expanded ex vivo with cytokines to enhance cytotoxicity and allow for tumor cell recognition in an HLA-unrestricted manner [206]. The combination regimens of CIK cells with pembrolizumab, IL-2, and sorafenib each demonstrated efficacy in mRCC case reports [207,208]. Additionally, DC vaccine/CIK cell hybrids (DC-CIKs) have been studied in combination with pembrolizumab and TKIs in advanced RCC with encouraging preliminary results [209,210]. DC vaccine and CIK cell co-administration also exhibited feasibility in a phase I/II study of advanced RCC [211,212].

## 5. Conclusions

The management of renal cell carcinoma has evolved significantly with the advent of immune checkpoint inhibitors and targeted therapies, offering improved outcomes for patients with clear cell RCC. However, variant RCC histopathologies remain challenging to treat, underscoring the need for novel therapeutic strategies. Recent advances in molecular profiling, biomarker-driven approaches, and emerging therapies provide hope for addressing these challenges. To fully realize these advancements, future efforts should prioritize inclusive clinical trial designs that account for the diversity of RCC subtypes and patient populations, including those historically underrepresented in research. Integrating real-world data and leveraging cutting-edge technologies such as multi-omics, artificial intelligence, and liquid biopsy assays may further refine treatment strategies and improve personalization of care.

## Figures and Tables

**Table 1:** Summary of immunotherapy trial results in variant histology cell renal cell carcinomas.

ICI Monotherapy					
Trial	Phase	Setting	Population	Treatment	Results
AcSé	II	1st line or later	50 patients with unresectable locally advanced or metastatic nccRCC	Nivolumab	Median follow-up 10.4 mo. 12-week ORR 6% (3 PR) SD rate 49% PD rate 44% mPFS 3.9 mo. (95% CI, 2.9–8.3) 12-month OS rate 47.7% (95% CI, 33.5–67.8) [18]
CheckMate 374	IIIb/IV	2nd line or later	44 patients with advanced nccRCC	Nivolumab	Median follow-up 11 mo. (range, 0.4–27) ORR 13.6% (95% CI, 5.2–27.4) 1 CR (chromophobe) 5 PR (2 papillary, 1 chromophobe, 1 collecting duct, 1 unclassified) mPFS 2.2 mo. (95% CI, 1.8–5.4) mOS 16.3 mo. (95% CI, 9.2-NE) [19]
KEYNOTE-427	II	1st line	165 patients with advanced nccRCC	Pembrolizumab	ORR 26.7%. Median DOR 29.0 mo. mPFS 4.2 mo. (95% CI, 2.9–5.6) mOS 28.9 mo. (95% CI, 24.3-NR) [20]
GU16-260 (Cohort B)	II	1st line	35 patients with advanced nccRCC	Nivolumab, optional salvage Nivolumab + Ipilimumab	ORR 14.3% (95% CI, 4.8–30.3%) ORR by histology: ⚬ Papillary: 5% ⚬ Chromophobe: 17% ⚬ Unclassified: 30% CR rate 5.7% PR rate 8.6% mPFS 4.0 mo. (range, 2.7–4.3). 2/5 responders have progressed [21]
ANZUP 1602 (Part 1)	II	ICI-naïve	83 patients with metastatic or unresectable nccRCC	Part 1: Nivolumab	Median follow-up 22 mo. (range, 16–30) OTRR was 16.9% (95% CI, 9.5–26.7), Median DOR 20.7 mo. (95% CI, 3.7-NR) mPFS 4.0 mo. (95% CI, 3.6–7.4) [22]
**TKI Monotherapy**					
**Trial**	**Phase**	**Setting**	**Population**	**Treatment(s)**	**Results**
SWOG S0317	II	1st line	45 patients with locally advanced or metastatic papillary RCC	Erlotinib	Overall RR 11% (95% CI, 3–24), DCR 64% mOS 27 mo. (95% CI, 13–36) [23]
NCT00726323	II	1st or 2nd line	74 patients with metastatic papillary RCC	Foretinib	ORR by Response Evaluation Criteria in Solid Tumors (RECIST) v1.0 13.5%, mPFS 9.3 mo. mOS NR [24]
CREATE	II	Relapsed/refractory	23 patients with advanced or metastatic type 1 papillary RCC	Crizotinib	MET+ cohort: ⚬ ORR 50% (95% CI, 6.8–93.2) ⚬ 12 mo. PFS 75% (95% CI, 12.8–96.1) ⚬ 12 mo. OS 75% (95% CI, 12.8–96.1) MET− cohort: ⚬ ORR: 6.3% (95% CI, 0.2–30.2) ⚬ 12 mo. PFS 27.3%, (95% CI, 8.5–50.4) 12 mo. OS 71.8%, (95% CI, 41.1–88.4) [25]
AXIPAP	II	1st line	44 patients with locally advanced or metastatic papillary RCC	Axitinib	Median follow-up 32.0 mo. (range, 13.1–39.9) 24-week PFR 45.2% (95% CI, 32.6%–∞) ORR 28.6% (95% CI, 15.7–44.6%) ⚬ Type 1 pRCC: 7.7% ⚬ Type 2 pRCC: 35.7% mPFS 6.6 mo. (95% CI, 5.5–9.2) ⚬ Type 1 pRCC: 6.7 mo. (95% CI, 5.5–9.2) ⚬ Type 2 pRCC: 6.2 mo. (95% CI, 5.4–9.2) mOS 18.9 mo. (95% CI, 12.8-NR) [26]
SAVOIR	III	1st or 2nd line	60 patients with *MET*-driven metastatic papillary RCC	Savolitinib vs. sunitinib	PFS HR 0.71 (95% CI, 0.37–1.36; *p* = 0.31) OS HR 0.51 (95% CI, 0.21–1.17, *p* = 0.11) [27]
BONSAI	II	1st line	23 patients with metastatic collecting duct RCC	Cabozantinib	ORR 35% (95% CI, 16–57%) mPFS 4 mo. (95% CI, 3–13) mOS 7 mo. (95% CI, 3–31) [28]
CABOSUN II ^a^	II	1st line	32 patients with advanced nccRCC	Cabozantinib vs. sunitinib	Median follow-up 33.3 mo. mPFS cabozantinib vs. sunitinib 8.2 vs. 13.8 mo. (1-sided *p* = 0.96) No statistically significant differences in ORR or OS between arms [29]
ANZUP 1802	II	ICI-ineligible or refractory	33 patients with locally advanced or metastatic nccRCC	Cabozantinib	PR rate 22% ⚬ 7/24 pts. with prior ICI and 0/7 pts. unsuitable for ICI Median treatment duration 7.5 cycles (range, 2–12) in pts. with prior ICI treatment, and 11 cycles (range, 2–12) in pts. unsuitable for ICI 90% of pts required dose reduction [30]
PAPMET ^b^	II	1st or 2nd line	147 patients with advanced papillary RCC	Cabozantinib, crizotinib, or savolitinib vs. sunitinib	Median follow-up 17.5 mo. mOS 21.5 mo. (95% CI, 12.0–28.1) with cabozantinib and 17.3 mo. (95% CI, 12.8–21.8) with sunitinib (HR = 0.83; 95% CI, 0.51–1.36; *p* = 0.46) [12]
**Combination Therapy**					
**Trial**	**Phase**	**Setting**	**Population**	**Treatments**	**Results**
SWOG S1107 ^c^	II	1st or 2nd line	50 patients with locally advanced or metastatic papillary RCC	Tivantinib ± erlotinib	ORR 0% mPFS arms 1 and 2: 2.0 and 3.9 mo., respectively OS arms 1 and 2: 10.3 and 11.3 mo., respectively [31]
NCT02724878	II	ICI-naïve	60 patients with metastatic RCC with variant histology and/or sarcomatoid features	Bevacizumab + atezolizumab	ORR 26% mPFS 8.3 mo. (95% CI, 5.7–10.9) PD-L1+ ORR 60% vs. 19% in PD-L1- [32]
CheckMate 920	IIIb/IV	1st line	52 patients with advanced or metastatic nccRCC	Nivolumab + ipilimumab	2 CR (1 papillary, 1 unclassified) 7 PR (4 papillary, 3 unclassified) 17 SD Median TTR 2.8 mo. (range, 2.1–14.8) Median DOR NR (range, 0.0+–27.8+) mPFS 3.7 mo. (95% CI, 2.7–4.6) mOS 21.2 mo. (95% CI, 16.6-NE) [33]
COSMIC-021	Ib/II	1st or 2nd line	32 patients with advanced nccRCC	Cabozantinib + atezolizumab	Median follow-up 37.2 mo. (range, 32.1–58.5) Investigator-evaluated ORR 31% (all PRs) DCR 94% (95% CI, 79–99) Median DOR 8.1 mo. (95% CI, 2.4–18.1) mPFS 9.3 mo. (95% CI, 5.5–12.3) mOS NR (95% CI, 23.0-NE) [34]
CALYPSO	I/II	VEGF treatment naïve or treatment refractory	41 patients with metastatic papillary RCC	Durvalumab + savolitinib	CR rate: ⚬ Overall population: 29% (95% CI, 16–46) ⚬ *MET*-driven cohort: 53% (95% CI, 28–77) ⚬ PD-L1+ cohort: 33% (95% CI, 17 to 54) mPFS 4.9 mo. (95% CI, 2.5–10.0) in the treated population and 12.0 mo. (95% CI, 2.9 to 19.4) in *MET*-driven pts. mOS 14.1 mo. (95% CI, 7.3 to 30.7) in the treated population and 27.4 mo. (95% CI, 9.3-NR) in *MET*-driven pts. [35]
ANZUP 1602 (Part 2)	II	ICI-naïve	41 patients with metastatic or unresectable nccRCC refractory to Nivolumab	Part 2: Salvage nivolumab + ipilimumab (up to 4 doses)	OTRR 10% Median DOR 13.5 mo. (95% CI, 4.8–19.7) mPFS 2.6 mo. (95% CI, 2.2–3.8) mOS 24 mo. (95% CI, 16–28) from time of enrolment in Part 1 [22]
KEYNOTE-B61	II	1st line	158 patients with advanced nccRCC	Lenvatinib + pembrolizumab	Median follow up 22.8 mo. (range, 16.6–27.6). ORR 51% (95% CI, 43–59) CR rate 8% PR rate 42% DCR 82% (95% CI, 75–88) Median DOR 19.5 mo. (range, 15.3-NR) mPFS 17.9 mo. (95% CI, 15.1–22.1) OS NR (95% CI, NR-NR) [13]
CA209-9KU	II	1st or 2nd line	40 patients with advanced or metastatic nccRCC	Cabozantinib + nivolumab	Median follow-up 34 mo. ORR 48% (95% CI, 31.5–63.9%) mPFS 13 mo. (95% CI, 7–16) 12-mo. and 24-mo. PFS rates 51% (95% CI, 34–65%) and 23% (95% CI, 11–37%), respectively mOS 28 mo. (95% CI 23–43) [14]
SUNNIFORECAST	II	1st line	309 patients with advanced nccRCC	Nivolumab + ipilimumab vs. SOC	Overall population 12 mo. OS rate 82.5% 12 mo. OS rate of Ipi/Nivo vs. SOC: 86.9% (95% CI, 80.2–91.5) vs. 76.8% (95% CI, 68.6–83.1; *p* = 0.014) mOS 42.4 mo. vs. 33.9 mo. ORR 25.4% (95% CI, 15.8–37.1) vs. 23.3% (95% CI, 15.1–33.4) mPFS 5.09 mo. (95% CI, 2.91–6.05) vs. 5.55 mo. (95% CI, 5.29–7.21) (not statistically significant) [36]

CI: confidence interval; CR: complete response; DOR: duration of response; DCR: disease control rate; ICI: immune checkpoint inhibitor; Ipi: ipilimumab; mo: months; mOS: median overall survival; mPFS: median progression-free survival; NE: not estimable; Nivo: nivolumab; NR: not reached; ORR: objective response rate; OTRR: objective tumor response rate; PFS: progression-free survival; PFR: progression-free rate; PR: partial response; pts: patients; RR: relative risk; SOC: standard-of-care; SD: stable disease; TTR: time-to-response; TKI: tyrosine kinase inhibitor. ^a^ Trial terminated early due to a change in standard therapy for PRCC unrelated to trial results. ^b^ Savolitinib and crizotinib arms were closed due to futility. ^c^ Trial terminated early due to futility.

**Table 2:** Summary of results from the first-line trials in variant histology renal cell carcinomas categorized by histologic subtype.

Trial	Phase	Population	Treatment(s)	Results by Histologic Subtype
KEYNOTE-427	II	165 patients with advanced nccRCC Papillary: 118 (71.5%) Chromophobe: 21 (12.7%) Unclassified: 26 (15.8%)	Pembrolizumab	ORR (95% CI): ⚬ Papillary: 28.8% (20.8–37.9) ⚬ Chromophobe: 9.5% (1.2–30.4) ⚬ Unclassified: 30.8% (14.3–51.8) CR (%): ⚬ Papillary: 7 (5.9%) ⚬ Chromophobe: 1 (4.8%) ⚬ Unclassified: 3 (11.5%) mPFS (95% CI): ⚬ Papillary: 5.5 (3.9–6.9) ⚬ Chromophobe: 3.9 (2.6–6.9) ⚬ Unclassified: 2.8 (2.8–5.1) mOS (95% CI): ⚬ Papillary: 31.5 (25.5-NR) ⚬ Chromophobe: 23.5 (9.3-NR) ⚬ Unclassified: 17.6 (7.5-NR) [20]
KEYNOTE-B61	II	158 patients with advanced nccRCC: Papillary: 93 (59%) Chromophobe: 29 (18%) Unclassified: 21 (13%) Translocation: 6 (4%) Other: 9 (6%)	Lenvatinib + pembrolizumab	ORR (95% CI): ⚬ Papillary: 54% (43–64) ⚬ Chromophobe: 35% (18–54) ⚬ Unclassified: 50% (27–73) ⚬ Translocation: 67% (22–96) ⚬ Other: 60% (26–88) CR (%): ⚬ Papillary: 10 (11%) ⚬ Chromophobe: 0 ⚬ Unclassified: 0 ⚬ Translocation: 1 (17%) ⚬ Other: 2 (20%) mPFS (95% CI) ⚬ Papillary: 17.5 (15-NR) ⚬ Chromophobe: 12.5 (3.9-NR) mOS: ⚬ Not reached [13,37]
CA209-9KU	II	40 patients with advanced or metastatic nccRCC: Papillary: 32 (80%) Unclassified without papillary features: 6 (15%) Translocation-associated: 2 (5%)	Cabozantinib + nivolumab	ORR (95% CI): ⚬ Papillary: 47% (30–64) ⚬ Unclassified without papillary features: 50% (12–88) ⚬ Translocation-associated: 50% (1–99) CR (%): ⚬ Papillary: 1 (3.1%) ⚬ Unclassified without papillary features: 0 ⚬ Translocation-associated: 0 mPFS (95% CI): ⚬ Papillary: 13 (7–16) ⚬ Unclassified without papillary features: 8 (1–NE) ⚬ Translocation-associated: 14 (5–23) [14]
PAPMET	II	44 patients with advanced papillary RCC	Cabozantinib	ORR 23% mPFS 9.0 mo. mOS: 21.5 mo. (95% CI, 12.0–28.1) [12]
PAPMET	II	46 patients with advanced papillary RCC	Sunitinib	ORR 4% mPFS 5.6 mo. mOS 17.3 mo. (95% CI, 12.8–21.8) [12]

CI: confidence interval; CR: complete response; mo: months; mOS: median overall survival; mPFS: median progression-free survival; NE: not estimable; NR: not reached; ORR: objective response rate; OS: overall survival; PFS: progression-free survival.

**Table 3:** Summary of phase III ICI-based combination therapy trial results in renal cell carcinoma with sarcomatoid features.

Trial	Phase	Setting	Treatments	Results
KEYNOTE-426	III	1st line	Pembrolizumab + axitinib vs. sunitinib	ORR 58.8% (95% CI, 44.2–72.4) vs. 31.5% (19.5–45.6) mPFS NR vs. 8.4 mo. (HR = 0.54; 95% CI, 0.29–1.00) OS HR = 0.58 (95% CI, 0.21–1.59) CR rate 11.8% (95% CI, 4.4–23.9) vs. 0% (0.0–6.6) [98]
CheckMate 214	III	1st line	Nivolumab + ipilimumab vs. sunitinib	ORR 60.8% vs. 23.1% mPFS 26.5 mo. vs. 5.1 mo. (HR = 0.54; 95% CI, 0.33–0.86) mOS NR vs. 14.2 mo. (HR = 0.45; 95% CI, 0.3–0.7) CR rate 18.9% vs. 3.1% [99]
CheckMate 9ER	III	1st line	Nivolumab + cabozantinib vs. sunitinib	ORR 55.9% vs. 22.0% mPFS 10.9 mo. vs. 4.2 mo. (HR = 0.39; 95% CI, 0.22–0.70) mOS NR vs. 19.7 mo. (HR = 0.36; 95% CI, 0.16–0.82) [100]
JAVELIN RCC 101	III	1st line	Avelumab + axitinib vs. sunitinib	ORR 47% vs. 21% PFS 7.0 vs. 4.0 mo. (HR = 0.57; 95% CI, 0.32–1.00) CR rate 4.3% vs. 0% [101]
ImMotion151	III	1st line	Atezolizumab + bevacizumab vs. sunitinib	ORR 49% vs. 14% PFS 8.3 vs. 5.3 mo. (HR = 0.52; 95% CI, 0.34–0.79) CR rate 10% vs. 3% [102]
CLEAR	III	1st line	Lenvatinib + pembrolizumab vs. sunitinib	ORR 60.7% vs. 23.8% (OR 8.85; 95% CI, 2.07–37.84) mPFS 11.1 mo. vs. 5.5 mo. (HR 0.39; 95% CI, 0.18–0.84) OS HR = 0.91 (95% CI, 0.32–2.58) mOS NR in either arm CR rate 10.7% vs. 0% [103]

CI: confidence interval; CR: complete response; HR: hazard ratio; mo: months; mOS: median overall survival; mPFS: median progression-free survival; NR: not reached; OS: overall survival; OR: odds ratio; ORR: objective response rate, PFS: progression-free survival.

**Table 4:** Summary of ICI-based trial results in non-clear cell renal cell carcinoma with sarcomatoid features.

Monotherapy				
Trial	Phase	Setting	Treatment	Results
KEYNOTE-427	II	1st line	Pembrolizumab	ORR 26.4% (95% CI, 16.5–38.1%) mPFS 4.2 mo. (95% CI, 2.9–5.6 mo.) mOS 18.7 mo. (95% CI, 13.0–24.8 mo.) PR 19.8% (95% CI, 11.2–30.9%) CR rate 6.6% (95% CI, 2.2–14.6%) [20]
**Combination Therapy**				
**Trial**	**Phase**	**Setting**	**Treatments**	**Results**
NCT02724878	II	1st line	Atezolizumab + bevacizumab	ORR 26% [32]
CheckMate 920	IIIb/IV	1st Line	Nivolumab + ipilimumab	ORR 41.1% (95% CI, 28.1–55.0%) mPFS 7.0 mo. (95% CI, 4.0–11.0 mo.) mOS 24.0 mo. (95% CI, 14.0-NE) PR rate 29.5% (95% CI, 18.0–43.6%) CR rate 11.6% (95% CI, 4.4–23.4%) [33]

CI: confidence interval; CR: complete response; mo: months; mOS: median overall survival; mPFS: median progression-free survival; NE: not estimable; ORR: objective response rate, PFS: progression-free survival.

**Table 5:** Table of ongoing and forthcoming clinical trials for variant histology renal cell carcinomas.

NCT Number	Histology	Treatment(s)	Phase	Status
NCT05347212	Renal medullary carcinoma	Nivolumab + relatimab	II	Active, not recruiting
NCT05286801	Renal medullary carcinoma and *SMARCB1* or *SMARCA4* deficient tumors	Tiragolumab + atezolizumab	II	Recruiting
NCT06161532	Renal medullary carcinoma	Sacituzumab govitecan ± atezolizumab	II	Recruiting
NCT06302569	Renal medullary carcinoma or collecting duct RCC	Enfortumab vedotin (EV) + pembrolizumab	II	Not yet recruiting
NCT05620134	Papillary RCC	JK08 (IL-15 antibody fusion protein targeting CTLA-4)	I/II	Active, not recruiting
NCT03866382	Papillary, chromophobe, sarcomatoid RCC and renal medullary carcinoma	Nivolumab + ipilimumab + cabozantinib	II	Recruiting
NCT05043090	*MET*-driven papillary RCC	Salvolitinib + durvalumab vs. sunitinib or durvalumab monotherapy	III	Recruiting
NCT04981509	Papillary RCC or HLRCC	Bevacizumab + erlotinib + atezolizumab	II	Recruiting
NCT05752552	*MET*-driven disease, hereditary renal papillary RCC	DO2-deuterated MET kinase inhibitor	I	Recruiting
NCT05678673	Papillary, unclassified (NOS), *MiT* translocation	XL092 (TKI) + nivolumab vs. sunitinib	III	Recruiting
NCT05411081	Papillary RCC	Cabozantinib vs. cabozantinib + atezolizumab	II	Recruiting
NCT05665361	Papillary RCC	Palbociclib + sasanlimab	I/II	Recruiting
NCT03595124	TFE/translocation RCC	Arm A: axitinib + nivolumab Arm B: axitinib monotherapy Arm C: nivolumab monotherapy	II	Active, not recruiting
NCT03388632	Open to all solid tumors	Arm 1: IL-15 + nivolumab Arm 2: IL-15 + ipilimumab Arm 3: IL-15 + nivolumab and Ipilimumab	I	Recruiting
NCT04071223	Non-clear cell and clear cell RCC with metastatic bone disease	Cabozantinib + radium-223	II	Recruiting
NCT05220267	Papillary, chromophobe, collecting duct carcinoma, renal medullary carcinoma, or unclassified	Anlotinib + sintilimab	II	Not yet recruiting
NCT05768464	Non-clear cell RCC (excluding chromophobe and eosinophilic RCC)	Toripalimab + axitinib	II	Recruiting
NCT05831891	Non-clear cell RCC	Fruquintinib and serplulimab	II	Not yet recruiting
NCT05808608	Non-clear cell RCC, sarcomatoid RCC	AK104 (PD-1/CTLA-4 bispecific antibody) + Axitinib	I/II	Not yet recruiting
NCT06053658	Non-clear cell RCC (excluding RMC and collecting duct)	Tivozanib + nivolumab	II	Recruiting

NCT: national clinical trial; NOS: not otherwise specified; HLRCC: hereditary leiomyomatosis and renal cell carcinoma; RMC: renal medullary carcinoma; TKI: tyrosine kinase inhibitor.
